# Awareness and interest in cannabis use for cancer management among cancer survivors

**DOI:** 10.1002/cam4.6902

**Published:** 2024-01-05

**Authors:** Ikponmwosa Osaghae, Rajesh Talluri, Onyema Greg Chido‐Amajuoyi, Kimberson Tanco, Dimpy P. Shah, Mala Pande, Sanjay Shete

**Affiliations:** ^1^ Department of Epidemiology The University of Texas MD Anderson Cancer Center Houston Texas USA; ^2^ Department of Biostatistics The University of Texas MD Anderson Cancer Center Houston Texas USA; ^3^ Division of Cancer Prevention and Population Sciences The University of Texas MD Anderson Cancer Center Houston Texas USA; ^4^ Department of Data Science University of Mississippi Medical Center Jackson Mississippi USA; ^5^ Department of Palliative, Rehabilitation and Integrative Medicine The University of Texas MD Anderson Cancer Center Houston Texas USA; ^6^ Mays Cancer Center, UT Health San Antonio MD Anderson Cancer Center San Antonio Texas USA; ^7^ Department of Gastroenterology, Hepatology and Nutrition The University of Texas MD Anderson Cancer Center Houston Texas USA

**Keywords:** cancer management, cancer survivors, cannabis awareness, cannabis interest, cannabis use

## Abstract

**Background:**

We examined the awareness, interest, and information sources relating to cannabis use for cancer management (including management of cancer symptoms and treatment‐related side effects) and determined factors associated with cancer survivors' awareness and interest in learning about cannabis use for cancer management.

**Methods:**

This was a cross‐sectional study of adult cancer survivors (*N* = 1886) receiving treatment at a comprehensive cancer center. Weighted prevalence and multivariable logistic regression analyses were conducted.

**Results:**

Among cancer survivors, 88% were aware and 60% were interested in learning about cannabis use for cancer management. Common sources of information to learn about cannabis use for cancer management were cancer doctors/nurses (82%), other patients with cancer (27%), websites/blogs (26%), marijuana stores (20%), and family/friends (18%). The odds of being aware of cannabis use for cancer management was lower among male compared to female survivors (adjusted odds ratio [AOR]: 0.61; 95% confidence interval [CI]: 0.41–0.90), non‐Hispanic Blacks compared to non‐Hispanic Whites (AOR: 0.36; 95% CI: 0.21–0.62), and survivors who do not support the legalization of cannabis for medical use compared to those who do (AOR: 0.10; 95% CI: 0.04–0.23). On the other hand, the odds of being interested in cannabis use for cancer management was higher among non‐Hispanic Blacks compared to non‐Hispanic Whites (AOR: 1.65; 95% CI: 1.04–2.62), and among cancer survivors actively undergoing cancer treatment compared to patients on non‐active treatment (AOR: 2.25; 95% CI: 1.74–2.91).

**Conclusion:**

Awareness of cannabis use for cancer management is high within the cancer survivor population. Results indicated health care providers are leading information source and should receive continued medical education on cannabis‐specific guidelines. Similarly, tailored educational interventions are needed to guide survivors on the benefits and risks of cannabis use for cancer management.

## 1 BACKGROUND

In 2022, there were over 18 million cancer survivors alive in the United States^1^ This number is expected to rise with advances in the early detection and treatment of cancer and a growing and aging United States population.^1^ Even with breakthroughs in cancer treatment and research, there is still no known cure for some cancers.^2^, ^3^, ^4^ Cancer management often involves a combination of activities beyond curative treatment including investigation and patient workup, palliation (treatment not aimed at achieving a cure, e.g., management of cancer‐related symptoms or treatment‐related side effects), curative therapy (treatment aimed at achieving a cure), and rehabilitation. Patients with cancer often experience symptoms related to their illness or chemotherapy, including pain, neuropathy, nausea, vomiting, anorexia, anxiety, depression, and sleep disorders.^5^, ^6^, ^7^, ^8^, ^9^ Moreover, cancer and cancer treatments have undesirable impacts on survivors' quality of life.^10^, ^11^, ^12^ Current treatments for most of the cancer and treatment‐related symptoms above are not always effective and can cause side effects and dependence.^13^, ^14^ This treatment dilemma necessitates novel approaches for managing cancer‐related symptoms and improving survivors' quality of life and outcome.

The use of cannabis (marijuana) and its derivatives for managing cancer and chemotherapy‐related symptoms is gaining popularity among survivors.^15^, ^16^ While the role of cannabis in cancer management is an area of ongoing research, there is evidence to support the use of cannabis or cannabis‐derived products as alternatives for alleviating several cancer and cancer treatment‐related symptoms.^17^, ^18^, ^19^ A systematic review and meta‐analysis found moderate‐quality evidence supporting the use of cannabinoids for treating chronic pain and spasticity, as well as low‐quality evidence for weight loss, nausea, vomiting, and sleep disorders.^19^ Also, several studies provide evidence to support the opioid sparing effects of cannabinoids which minimizes potential adverse events and dependence on opioids for chronic pain management.^20^, ^21^, ^22^ More so, the 2017 American Society for Clinical Oncology antiemetic guidelines recommend cannabinoids, dronabinol, or nabilone in patients not responsive to conventional antiemetics.^23^

In the United States, cannabis is legalized for medical purposes in 38 states and the District of Columbia (DC) and legalized for recreational purposes in 23 states and DC.^24^ Cannabis laws in Texas are restrictive, only permitting cannabis products with low tetrahydrocannabinol levels for medicinal purposes and in limited situations.^25^ Despite conflicting reports on the benefits of cannabis for cancer management and ongoing controversies around its legalization, cancer survivors continue to use cannabis to alleviate their symptoms even without guidance from their health care providers.^15^, ^26^, ^27^ Access to unregulated cannabis products may increase the risk of side effects and overdose, as well as interactions with other medications.^26^ There is a persistent knowledge gap in the awareness of and interest in cannabis use for cancer management among survivors due to the absence of systematic studies evaluating their prevalence. Past studies have focused on lifetime or past cannabis use by patients with cancer, not necessarily on its use for cancer management, and have concentrated on a subpopulation of survivors.^15^, ^16^, ^28^ Also, these prior studies were based on relatively small sample sizes of cancer survivors. Moreover, given the frequent exposure of survivors to untrusted information sources, it is important to understand the sources of information about cannabis use for cancer management that cancer survivors use and to determine the factors that influence their awareness and interest in learning about cannabis use for managing their symptoms.^29^ This insight can help guide interventions to educate cancer survivors on safe and effective cannabis use. In addition, sociodemographic differences in survivors' symptom burden necessitate an understanding of how a survivor's sociodemographic factors affect their awareness and interest in cannabis use.^30^, ^31^ For instance, it has been shown that being young, male, Black, and of low income and educational status is associated with a higher frequency of cannabis use in the general population.^32^ More so, tobacco use and smoking have been associated with the use of cannabis.^33^, ^34^ Therefore, it is pertinent to determine how cancer survivors' awareness and interest in cannabis use for cancer management differ by their sociodemographic characteristics, as beliefs and perceptions influence behaviors, and use patterns often vary by demographics. Amidst misinformation and disinformation on medicinal cannabis and the reports on patients using cannabis for therapeutic purposes without medical advice, our study would inform targeted educational interventions among survivors to optimize the benefits of cannabis use in cancer management while minimizing risks. We hypothesize that the awareness and interest in learning about cannabis use for cancer management differ across survivors' sociodemographic characteristics, perceptions, treatment status, and behavioral practices.

## 2 METHODS

### 2.1 Data source and study population

This cross‐sectional study uses data from a survey conducted by The University of Texas MD Anderson Cancer Center (MDACC) between November 2021 and October 2022. The target population was patients with cancer and survivors ≥18 years old at different stages of treatment and survivorship who are either actively undergoing treatment or have recently completed treatment within the past 5 years at MDACC. The survey instrument was adapted from previously published questionnaires and other validated tools that assessed cannabis use‐related topics among patients with cancer.^15^, ^35^, ^36^ The study participant selection was stratified by (1) sex (male/ female), (2) race (non‐Hispanic Black/ any other race), and (3) cancer treatment status (active/not active). We intentionally oversampled non‐Hispanic Blacks to increase the sample size and robustness of data for this subpopulation. Eligible participants received an email invitation with a link to complete either the English or Spanish version of the survey in REDCap (https://www.project‐redcap.org/). All participants provided informed consent, and the survey took about 15 min to complete. The survey responses were anonymized, such that the stored survey responses could not be linked to any personal identifiers including the participants' email. Furthermore, to maintain anonymity, participants were not sent follow‐up email reminders. The study approval was provided by The University of Texas MD Anderson Cancer Center Ethical Review Board. Reporting of this study followed the Strengthening the Reporting of Observational Studies in Epidemiology guidelines.

### 2.2 MEASURES

#### 2.2.1 Dependent variables

##### 2.2.1.1 Awareness of cannabis use for cancer management

The awareness of cannabis use for cancer management (cannabis use to manage cancer symptoms or treatment‐related side effects) was assessed based on the survey question, “Have you ever heard of cannabis/marijuana use for cancer management? Examples of cannabis/marijuana products are CBD oil; pills, tinctures, concentrates or dry leaf cannabis/marijuana; cannabis creams and gels; and edible products like baked goods, candy, and beverages containing cannabis/marijuana.” Responses were yes/no.

##### 2.2.1.2 Interest in learning about cannabis use for cancer management

The interest in learning about cannabis use for cancer management (cannabis use to manage cancer symptoms or treatment‐related side effects) was assessed based on the survey question, “Are you interested in learning more about cannabis/marijuana use for cancer management?” Responses were Yes/No.

##### 2.2.1.3 Source of information on cannabis use for cancer management

This was assessed based on two separate survey questions. First, “Where WOULD you most likely go if you wanted to learn more about cannabis/marijuana use for cancer?” [Participants were allowed to select multiple sources from a list of possible sources]. Second, this was assessed using the question, “Which of the following sources of information are you most likely to TRUST for information about the use of cannabis/marijuana for cancer management?” [Participants selected their most likely source of information from the following list of possible sources]; “A cancer doctor or nurse involved in my cancer treatment,” “A doctor or nurse outside of my cancer team,” “Another cancer patient,” “Pamphlet or handout,” “Nutritionist,” “Naturopath/herbalist,” “Friend/family member,” “Medicinal cannabis/marijuana store,” “Newspaper/magazine article,” “TV/radio advertisement,” “Social media (Facebook, Twitter, etc.),” and “Websites or blogs,” and “Other sources.”

#### 2.2.2 Independent variables

##### 2.2.2.1 Cancer treatment status

Participants were asked, “Where are you in your cancer treatment?” Those who responded “Currently undergoing treatment” were classified as cancer survivors on active treatment. On the other hand, those who answered “Finished treatment and undergoing follow‐up/check‐up” or “Completed treatment and all follow‐ups,” or “Newly diagnosed, not yet started treatment” were classified as cancer survivors on non‐active treatment.

##### 2.2.2.2 Cigarette smoking

Smoking status was assessed based on the question, “Have you smoked at least 100 cigarettes in your entire life?” Those who answered “yes” were classified as ever‐smokers, while those who answered “no” were classified as never smokers.

##### 2.2.2.3 State cannabis law

The status of cannabis law in the state of residence of participants was categorized as fully legal (legalized for both medicinal and recreational use), legal for medicinal use only, and fully illegal (illegal for medicinal and recreational use).

##### 2.2.2.4 Support legalization for medical use

Support for the legalization of cannabis for medical use by patients with cancer was assessed using the question, “In your opinion, should marijuana/cannabis use for medicinal purposes be legalized throughout the US?” Possible responses were “yes,” “no,” and “unsure.”

##### 2.2.2.5 Sociodemographic characteristics

Sociodemographic factors assessed were age (<50 years, 50–64 years, and ≥65 years), sex (female/male), race/ethnicity (non‐Hispanic White, non‐Hispanic Black, non‐Hispanic Other, and Hispanic), education (high school/GED or lower, some college, college degree, and graduate degree), income (<$35,000, $35,000 to <$50,000, $50,000 to <$75,000, $75,000 to <$100,000, ≥$100,000), and residence (rural/urban). Participants' Rural–Urban Continuum Codes (RUCC) were determined by linking participants' zip codes obtained from electronic health records with the Federal Information Processing Standards. RUCC 1–3 were classified as urban, while RUCC 4–9 were classified as rural.

### 2.3 Analysis

The survey was stratified on sex, race, and cancer treatment status. Survey weights were constructed for each study participant to account for the stratified sampling design. The base sampling weights for each respondent were created using the inverse probability of being included in the survey. No poststratification adjustments were performed for any other covariate. We adjusted for nonresponse using a response propensity model in which the three stratifying variables were included in the propensity model.^37^ After estimating the response propensities from the model, the weights were then redistributed from the nonresponders to the responders based on these propensities. The final weights, including the adjustment for nonresponse, were then used to perform the statistical analyses. We estimated the prevalence of awareness and interest in learning about cannabis use for cancer management among survivors stratified by sociodemographic characteristics, perceptions, and behavioral practices of survivors. Also, we estimated the prevalence of various information sources utilized by survivors in learning about cannabis use for cancer management as well as their most trusted source of information. Lastly, we used multivariable logistic regression models to determine the odds of awareness of and interest in learning about cannabis use for cancer management among cancer survivors. Variables included in our analysis were selected a priori based on our hypothesis and the literature. Thus, no stepwise variable selection was conducted. The weight estimation and survey analyses were performed using the “survey” and “svrep” packages in R version 4.2.1. The prevalence and odds ratios reported in this study are all survey weighted. For all statistical analyses, *p*‐values were calculated using Wald test statistic, and significance was defined as a two‐sided *p* value ≤ 0.05.

## 3 RESULTS

A total of 1886 cancer survivors completed the survey, representing a response rate of 11.7%. Overall, the majority of participants were aged ≥65 years (47.7%), female (56.3%), non‐Hispanic White (84.5%), had a college degree (37.4%), earned ≥$100,000 per annum (52.0%), and were not receiving active cancer treatment (72.3%). Also, most of the participants resided in urban regions (87.3%), in states where cannabis is fully illegal (75.7%), and support the legalization of cannabis for medical use (80.4%) (Table 1).

**TABLE 1:** Prevalence of awareness and interest in cannabis use for cancer management by sociodemographic characteristics, perception, and behavioral practices of survivors (*N* = 1886).

Characteristics	Categories	Overall	Wt% [95% CI]			
			Awareness of cannabis use for cancer management		Interested in learning about cannabis use for cancer management	
			No	Yes	No	Yes
Age, years	<50	15.4 [13.3–17.8]	11.1 [7.0–17.2]	88.9 [82.8–93.0]	26.3 [19.8–34.1]	73.7 [65.9–80.2]
	50–64	36.9 [34.0–39.8]	9.4 [7.0–12.4]	90.6 [87.6–93.0]	38.0 [33.3–43.0]	62.0 [57.0–66.7]
	≥65	47.7 [44.8–50.7]	14.1 [11.6–17.0]	85.9 [83.0–88.4]	45.4 [41.3–49.6]	54.6 [50.4–58.7]
Sex	Female	56.3 [53.6–59.0]	9.9 [7.7–12.8]	90.1 [87.2–92.3]	41.6 [37.4–46.0]	58.4 [54.0–62.6]
	Male	43.7 [41.0–46.4]	15.3 [13.1–17.8]	84.7 [82.2–86.9]	38.3 [35.1–41.6]	61.7 [58.4–64.9]
Race/ethnicity	Non‐Hispanic White	84.5 [82.4–86.3]	11.5 [9.7–13.5]	88.5 [86.5–90.3]	42.1 [39.0–45.2]	57.9 [54.8–61.0]
	Non‐Hispanic Black	3.0 [2.5–3.5]	21.8 [15.5–29.6]	78.2 [70.4–84.5]	30.0 [22.9–38.3]	70.0 [61.7–77.1]
	Hispanic	8.1 [6.6–9.8]	12.1 [6.6–21.1]	87.9 [78.9–93.4]	27.8 [19.2–38.3]	72.2 [61.7–80.8]
	Other	4.5 [3.5–5.8]	21.5 [12.6–34.3]	78.5 [65.7–87.4]	34.5 [22.7–48.5]	65.5 [51.5–77.3]
Education	High school/GED or lower	9.3 [7.7–11.1]	11.1 [6.7–17.8]	88.9 [82.2–93.3]	37.4 [28.6–47.2]	62.6 [52.8–71.4]
	Some college	24.6 [22.3–27.2]	11.2 [8.1–15.2]	88.8 [84.8–91.9]	33.6 [28.4–39.3]	66.4 [60.7–71.6]
	College degree	37.4 [34.7–40.2]	12.4 [9.6–15.7]	87.6 [84.3–90.4]	41.1 [36.5–45.8]	58.9 [54.2–63.5]
	Graduate degree	28.7 [26.2–31.3]	13.7 [10.7–17.4]	86.3 [82.6–89.3]	46.0 [40.8–51.3]	54.0 [48.7–59.2]
Income, per annum	<$35,000	10.0 [8.4–11.8]	8.8 [5.2–14.6]	91.2 [85.4–94.8]	26.3 [19.0–35.2]	73.7 [64.8–81.0]
	$35,000 to <$50,000	8.7 [7.2–10.5]	17.3 [10.9–26.3]	82.7 [73.7–89.1]	37.2 [28.0–47.5]	62.8 [52.5–72.0]
	$50,000 to <$75,000	12.7 [10.8–14.8]	18.3 [12.7–25.6]	81.7 [74.4–87.3]	40.6 [32.6–49.2]	59.4 [50.8–67.4]
	$75,000 to <$100,000	16.7 [14.6–19.0]	11.3 [7.6–16.5]	88.7 [83.5–92.4]	35.7 [29.1–42.9]	64.3 [57.1–70.9]
	≥$100,000	52.0 [49.0–54.9]	10.9 [8.8–13.6]	89.1 [86.4–91.2]	41.8 [37.8–45.9]	58.2 [54.1–62.2]
Residence	Urban	87.3 [85.4–89.0]	12.1 [10.4–14.2]	87.9 [85.8–89.6]	39.7 [36.8–42.8]	60.3 [57.2–63.2]
	Rural	12.7 [11.0–14.6]	13.1 [9.0–18.9]	86.9 [81.1–91.0]	43.3 [35.8–51.1]	56.7 [48.9–64.2]
Cancer treatment status	Non‐active	72.3 [70.2–74.3]	11.4 [9.3–13.8]	88.6 [86.2–90.7]	44.3 [40.7–48.0]	55.7 [52.0–59.3]
	Active	27.7 [25.7–29.8]	14.6 [12.2–17.3]	85.4 [82.7–87.8]	29.4 [26.3–32.7]	70.6 [67.3–73.7]
State cannabis law	Fully legal	16.3 [14.3–18.6]	9.9 [6.5–14.9]	90.1 [85.1–93.5]	43.2 [36.2–50.5]	56.8 [49.5–63.8]
	Legal for medicinal use only	8.0 [6.6–9.8]	15.3 [9.2–24.2]	84.7 [75.8–90.8]	44.7 [34.5–55.3]	55.3 [44.7–65.5]
	Fully illegal	75.7 [73.1–78.1]	12.4 [10.4–14.6]	87.6 [85.4–89.6]	39.4 [36.2–42.7]	60.6 [57.3–63.8]
Support legalization for medical use	Yes	80.4 [78.0–82.5]	8.8 [7.3–10.7]	91.2 [89.3–92.7]	32.9 [29.9–36.0]	67.1 [64.0–70.1]
	No	2.3 [1.7–3.3]	47.3 [30.7–64.5]	52.7 [35.5–69.3]	91.5 [71.4–97.9]	8.5 [2.1–28.6]
	Unsure	17.3 [15.2–19.6]	23.1 [17.9–29.3]	76.9 [70.7–82.1]	67.5 [60.8–73.5]	32.5 [26.5–39.2]
Smoking status	No	59.9 [57.1–62.6]	14.3 [11.9–17.0]	85.7 [83.0–88.1]	46.1 [42.3–49.9]	53.9 [50.1–57.7]
	Yes	40.1 [37.4–42.9]	9.1 [7.1–11.7]	90.9 [88.3–92.9]	31.6 [27.7–35.7]	68.4 [64.3–72.3]

Abbreviations: CI, confidence interval; Wt%, weighted percentage; Wt_n, weighted number.

### 3.1 Awareness of cannabis use for cancer management

Of the survey respondents, 87.7% (95% confidence interval [CI]: 85.8%–89.4%) were aware of cannabis use for cancer management. The prevalence of awareness of cannabis use for cancer management was higher among females compared to males (90.1% vs. 84.7%) and highest among survivors aged 50–64 years (90.6%) compared to those <50 years (88.9%), and ≥65 years (85.9%). Also, the prevalence of awareness of cannabis use for cancer management was highest among non‐Hispanic Whites (88.5%) compared to Hispanics (87.9%), non‐Hispanic Other (78.5%), and non‐Hispanic Blacks (78.2%). In addition, the prevalence of awareness of cannabis use for cancer management was higher among survivors who support compared to those who do not support the legalization of cannabis for medical use (91.2% vs. 52.7%), and among ever‐smokers compared to never‐smokers (90.9% vs. 85.7%). Furthermore, the prevalence of awareness of cannabis use for cancer management was highest among survivors with high school/GED or lower education (88.9%), those earning <$35,000 per annum (91.2%), those residing in urban areas (87.9%), and in non‐active cancer treatment (88.6%) (Table 1).

Results of multivariable logistic regression analyses (Table 2) revealed that male survivors reported 39% lower odds (adjusted odds ratio [AOR]: 0.61; 95% CI: 0.41–0.90) of being aware of cannabis use for cancer management compared to females. Also, non‐Hispanic Blacks reported 64% lower odds (AOR: 0.36; 95% CI: 0.21–0.62) of being aware of cannabis use for cancer management compared to non‐Hispanic Whites. Compared to those in support, survivors not supporting the legalization of cannabis for medical use reported 90% lower odds (AOR: 0.10; 95% CI: 0.04–0.23) of being aware of cannabis use for cancer management. Also, ever‐smokers reported 58% higher odds (AOR: 1.58; 95% CI: 1.04–2.40) of awareness of cannabis use for cancer management than never‐smokers. However, survivor's age, education, income (per annum), residence, cancer treatment status, and state cannabis law were not significantly associated with awareness of cannabis use for cancer management (Table 2).

**TABLE 2:** Multivariable regression analysis of the association between sociodemographic characteristics, perception, and behavioral practices of survivors with awareness and interest in cannabis use for cancer management.

Variable name	Categories	Awareness of cannabis use for cancer management				Interested in learning about cannabis use for cancer management			
		Adjusted odds ratio	Lower 95% CI	Upper 95% CI	*p*‐Value	Adjusted odds ratio	Lower 95% CI	Upper 95% CI	*p*‐Value
Age, years	<50	Ref	Ref	Ref	Ref	Ref	Ref	Ref	Ref
	50–64	1.61	0.84	3.10	0.151	0.61	0.38	0.99	0.044
	≥65	1.01	0.55	1.87	0.967	0.45	0.28	0.72	0.001
Sex	Female	Ref	Ref	Ref	Ref	Ref	Ref	Ref	Ref
	Male	0.61	0.41	0.90	0.014	1.01	0.75	1.35	0.949
Race/ethnicity	Non‐Hispanic White	Ref	Ref	Ref	Ref	Ref	Ref	Ref	Ref
	Non‐Hispanic Black	0.36	0.21	0.62	<0.001	1.65	1.04	2.62	0.032
	Hispanic	0.56	0.26	1.20	0.136	1.69	0.90	3.16	0.103
	Other	0.55	0.22	1.38	0.202	1.38	0.63	3.04	0.424
Education	High school/GED or lower	Ref	Ref	Ref	Ref	Ref	Ref	Ref	Ref
	Some college	0.92	0.43	1.97	0.821	1.19	0.68	2.10	0.537
	College degree	0.71	0.34	1.48	0.361	1.10	0.63	1.91	0.738
	Graduate degree	0.64	0.30	1.35	0.239	0.93	0.53	1.64	0.813
Income, per annum	<$35,000	Ref	Ref	Ref	Ref	Ref	Ref	Ref	Ref
	$35,000 to <$50,000	0.78	0.31	1.94	0.596	1.28	0.63	2.60	0.504
	$50,000 to <$75,000	0.60	0.26	1.42	0.248	0.71	0.37	1.37	0.310
	$75,000 to <$100,000	0.78	0.34	1.78	0.558	0.93	0.51	1.72	0.823
	≥$100,000	1.04	0.50	2.18	0.91	0.69	0.39	1.19	0.182
Residence	Urban	Ref	Ref	Ref	Ref	Ref	Ref	Ref	Ref
	Rural	0.78	0.45	1.37	0.393	1.05	0.69	1.61	0.807
Cancer treatment status	Non‐active	Ref	Ref	Ref	Ref	Ref	Ref	Ref	Ref
	Active	0.89	0.63	1.26	0.505	2.25	1.74	2.91	<0.001
State cannabis law	Fully legal	Ref	Ref	Ref	Ref	Ref	Ref	Ref	Ref
	Legal for medicinal use only	0.63	0.25	1.62	0.34	0.96	0.53	1.72	0.882
	Fully illegal	0.61	0.33	1.15	0.129	1.19	0.80	1.79	0.391
Support legalization for medical use	Yes	Ref	Ref	Ref	Ref	Ref	Ref	Ref	Ref
	No	0.10	0.04	0.23	<0.001	0.02	0.00	0.11	<0.001
	Unsure	0.32	0.20	0.49	<0.001	0.24	0.17	0.35	<0.001
Smoking status	No	Ref	Ref	Ref	Ref	Ref	Ref	Ref	Ref
	Yes	1.58	1.04	2.40	0.032	1.83	1.36	2.47	<0.001

Abbreviations: CI, confidence interval; Ref, reference group.

### 3.2 Interest in learning about cannabis use for cancer management

Regarding interest in learning about cannabis use for cancer management, 59.8% (95% CI: 57.0%–62.6%) of survivors were interested in learning about cannabis use for cancer management. The prevalence of interest was higher among males compared to females (61.7% vs. 58.4%) and highest among survivors <50 years (73.7%), compared to those aged 50–64 years (62.0%), and ≥65 years (54.6%). Also, the prevalence of interest in learning about cannabis use for cancer management was highest among Hispanics (72.2%), compared to non‐Hispanic Blacks (70.0%), non‐Hispanic Other (65.5%), and non‐Hispanic White (57.9%). In addition, the prevalence of interest in cannabis use for cancer management was higher among survivors in active cancer treatment compared to those in non‐active cancer treatment (70.6% vs. 55.7%), those who support compared to those who do not support the legalization of cannabis for medical use (67.1% vs. 8.5%), and among ever‐smokers compared to never‐smokers (68.4% vs. 53.9%). Furthermore, the prevalence of interest in learning about cannabis use for cancer management was highest among survivors with some college education (66.4%), those earning <$35,000 per annum (73.7%), and higher among those residing in urban areas (60.3%) (Table 1).

Results of multivariable regression analysis (Table 2) showed that survivors aged 50–64 years and ≥65 years had 39% (AOR: 0.61; 95% CI: 0.38–0.99) and 55% (AOR: 0.45; 95% CI: 0.28–0.72) lower odds, respectively, of being interested in learning about cannabis use for cancer management compared to those <50 years. On the other hand, non‐Hispanic Blacks had 65% higher odds (AOR: 1.65; 95% CI: 1.04–2.62) of being interested in cannabis use for cancer management compared to non‐Hispanic Whites. Compared to those in support, survivors not in support of the legalization of cannabis for medical use had 98% lower odds (AOR: 0.02; 95% CI: 0.00–0.11) of being interested in learning about cannabis use for cancer management. Furthermore, cancer survivors undergoing active cancer management had 125% higher odds (AOR: 2.25; 95% CI: 1.74–2.91) of being interested in learning about cannabis use for cancer management than those in the non‐active treatment group. In addition, ever‐smokers had 83% higher odds (AOR: 1.83; 95% CI: 1.36–2.47) of interest in learning about cannabis use for cancer management compared to never‐smokers. Survivor's sex, education, income (per annum), residence, and state cannabis law were not significantly associated with interest in learning about cannabis use for cancer management (Table 2).

### 3.3 Source of information

**FIGURE 1:**
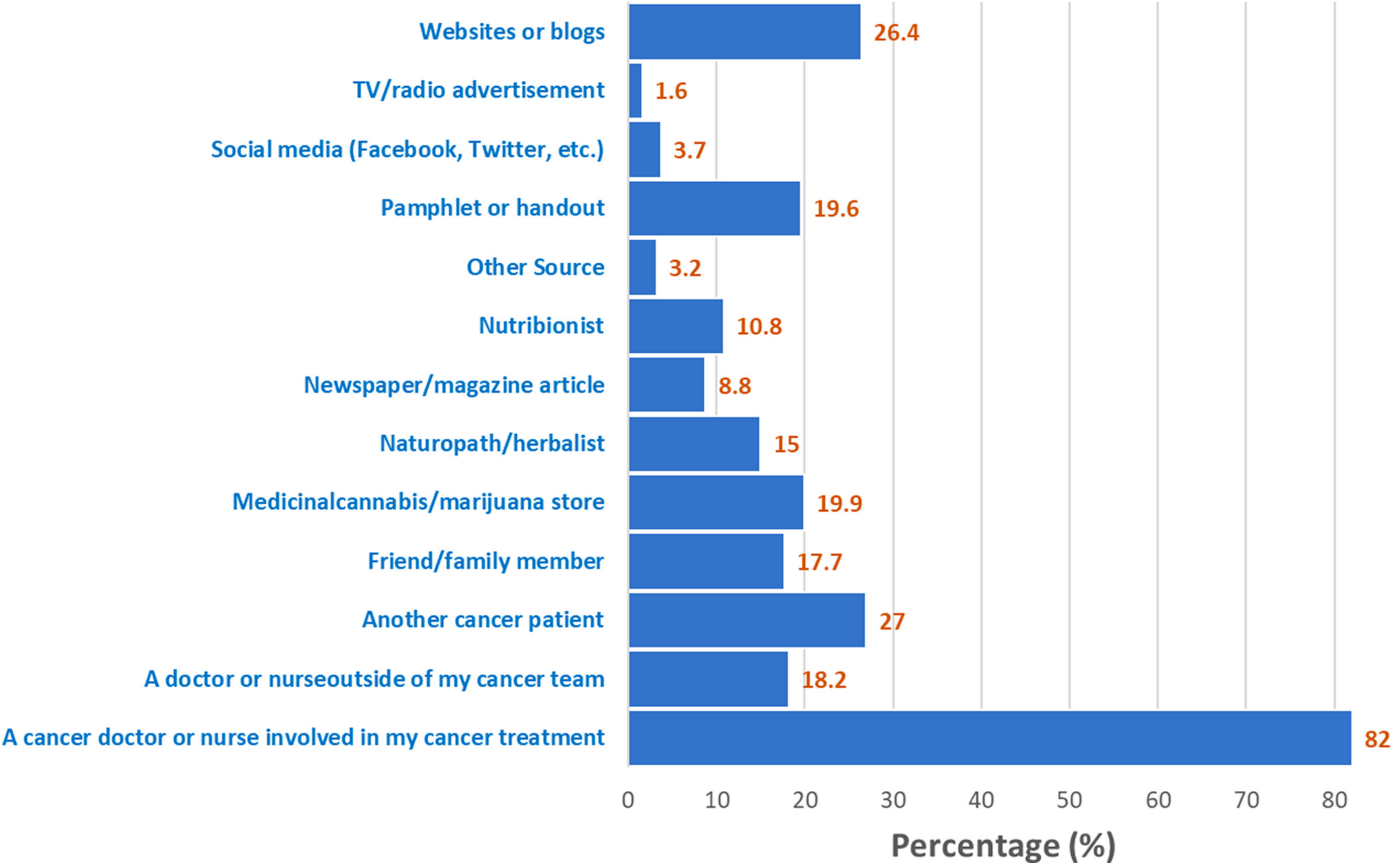
Cancer survivor's most likely source of information to learn more about cannabis use for cancer management.

**FIGURE 2:**
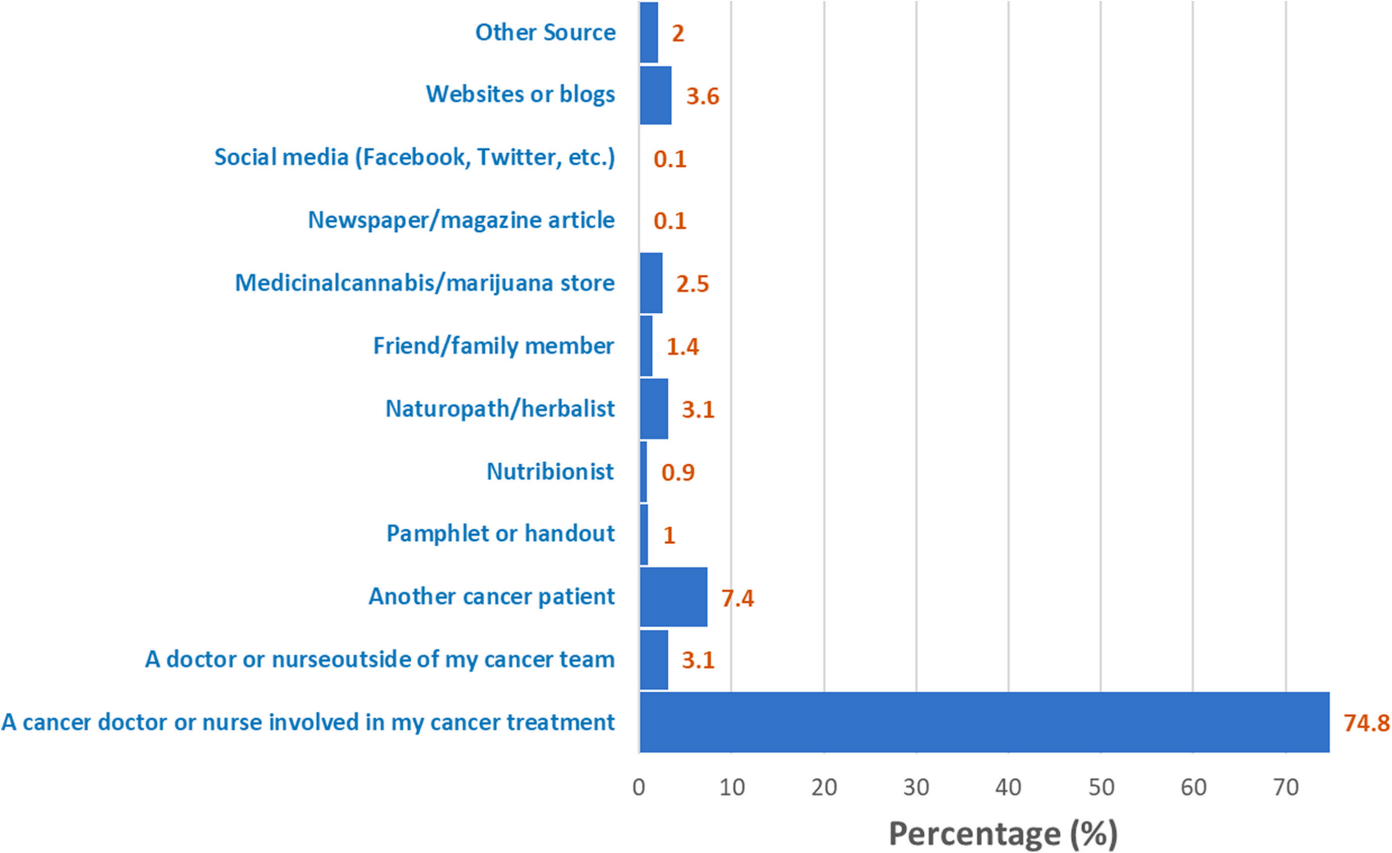
Cancer survivor's most trusted source of information about the use of cannabis for cancer management.

In terms of source of information (Figures 1 and 2), survivors were most likely to learn about cannabis use for cancer management from their cancer doctor or nurse involved in their treatment (82.0%) (Figure 1). However, we found that survivors also commonly sought such information from other patients with cancer (27.0%), websites or blogs (26.4%), pamphlets or handouts, (19.6%), marijuana stores (19.9%), and family/friends (17.7%). Furthermore, survivors' most trusted source of information on cannabis use for cancer management was their cancer doctor or nurse (74.8%), another patient with cancer (7.4%), and websites or blogs (3.6%) (Figure 2).

## 4 DISCUSSION

Overall, study findings revealed high levels of awareness and interest in cannabis use for cancer management among survivors at different stages of treatment at a large comprehensive cancer center. We found that whereas male survivors and non‐Hispanic Black survivors were less likely to be aware of cannabis use in cancer management, non‐Hispanic Black survivors were more likely to be interested in cannabis use in managing their cancer symptoms. In addition, survivors who do not support the legalization of cannabis for medical use were less likely to be aware of its use in cancer management while cancer survivors undergoing active cancer treatment were more likely to be interested in cannabis use in cancer management. Our findings of a high prevalence of awareness and interest in cannabis use in cancer management among survivors resonate and align with a previous study indicative of growing interest in the use of cannabinoids as a palliative for cancer management.^15^ Our study and that by Pergam et al. were conducted among adult cancer survivors receiving cancer treatment at an NCI‐designated cancer center. Whereas the study by Pergam et al. was in a state where cannabis is legalized for both medicinal and recreational use, our study was conducted in a state with restrictive cannabis laws. Importantly, findings from both studies indicate that in states with either liberal or restrictive cannabis laws, cancer survivors are interested in learning about cannabis use in managing their cancer symptoms. In another study focused on breast cancer survivors by Weiss et al., 41% of participants were interested in cannabis because they lacked alternative ways of treating their cancer symptoms.^16^ Our study, involving a larger sample of survivors, further highlights the growing interest in cannabis use as an alternative to manage cancer‐related symptoms. We also evaluated the sources of cannabis‐related information and factors associated with awareness of and interest in cannabis use for cancer management among patients with cancer/survivors. Patients with cancer have reported beneficial effects from cannabinoids and their analogs for cancer management or as a palliative for chemotherapy‐related symptoms.^16^, ^38^, ^39^ With legal limitations on access to cannabis in some states in the United States and controversies around its benefits, patients with cancer have been shown to use cannabis to treat various symptoms without telling their doctors.^16^ More so, most participants in our study were undergoing active cancer treatment, a phase known to be accompanied by chemotherapy‐related symptoms that are often unresponsive to conventional treatment. The need for alternative means of allaying these symptoms and improving survivors' overall quality of life during this phase may explain the high prevalence of interest and awareness in cannabis for medical management seen in our study.

Furthermore, we found that whereas most cancer survivors seek information on cannabis use for cancer management from their oncologist or nurse practitioner, a substantial percentage of survivors also seek such information from websites/blogs, cannabis stores, or family/friends. Most respondents in our study were from states where cannabis use is illegal for recreational and medicinal purposes, which may partly explain survivors' frequent utilization of unconventional and untrusted sources of information rather than their health care providers for cannabis‐related information. More so, other studies have shown that cancer survivors are more likely to seek health information from the internet or media before considering trusted sources such as their health care providers.^16^, ^29^ This is noteworthy given that social media, dispensaries, and family/friends have been associated with misinformation about cannabis.^40^ Although social media platforms may be useful in increasing awareness and educating survivors about cannabis use in cancer management, reliance on social media for information on cannabis use may expose survivors to misinformation and disinformation, resulting in risky health decisions.^41^ This underscores the need for clear policies to regulate and monitor misinformation and promotional activities related to cannabis within the social media space. Moreover, health care providers often lack sufficient knowledge to advice patients on the benefits of cannabis for cancer management.^42^ Also, the legal issues around cannabis and dissenting views on the benefits and risks of cannabis for medical purposes may limit health care professionals (HCPs) from discussing cannabis use with their patients. Findings from our study hint at the need for increased engagement of cancer survivors by their health care providers as a trusted source of information on the benefits of cannabis for cancer management. Given that over 70% of patients with cancer identify HCPs as their most trusted/likely source of information on cannabis use in cancer management, there is a need for cannabis‐related training for HCPs on up‐to‐date guideline‐based indications, benefits, and risks of cannabis use in cancer management. Also, more cannabis‐related studies and evidence‐based guidelines are needed to empower HCPs to care for survivors. In addition, cancer survivors should be educated on the benefits and risks of using cannabis as an alternative for managing cancer and chemotherapy‐related symptoms.

Non‐Hispanic Black survivors reported to be less aware of or interested in cannabis for cancer management than non‐Hispanic White survivors. This finding aligns with other studies which revealed that most medical cannabis patients are non‐Hispanic White.^43^, ^44^ Over the years, non‐Hispanic Blacks have been disproportionately arrested for non‐violent possession of cannabis, which is still a Schedule 1 drug in the United States^45^ The trauma from these experiences or sheer fear of arrest may account for their lack of interest in cannabis for medical use. In addition, our study revealed that survivors in support of cannabis legislation were more likely to be aware of and interested in cannabis use for cancer management. This is not surprising, given that beliefs tend to drive use patterns. Furthermore, we found that cancer survivors in active treatment were more likely to be interested in cannabis use for cancer management. This may be related to the fact that the active phase of cancer treatment is often accompanied by acute symptoms that are not readily amenable to conventional treatment.^14^, ^46^, ^47^ Socioeconomic factors such as the educational status and income of survivors were not associated with either awareness or interest in learning about cannabis use in cancer management. This finding may be attributed to the shared experience of symptoms from cancer and its treatment across different socioeconomic strata among survivors. Consequently, awareness and interest in exploring alternative medications, such as medicinal cannabis, to alleviate symptoms may persist at a comparable level regardless of the socioeconomic status of survivors.

Ever‐smokers were more aware of and interested in cannabis use for cancer management. Our finding resonates with the literature indicating an association between smoking and cannabis use in the general population.^34^ In a nationally representative study in the United States, daily cannabis use was higher among cigarette smokers and increased among youth and female cigarette smokers.^48^ It has been suggested that for inhalational cannabis products, co‐use with smoking could be related to the common route of administration.^49^ The growing popularity and interest in vaping, cigarette, and e‐cigarette use, coupled with their co‐use with cannabis products, may partly explain the growing awareness and interest in cannabis use for cancer management.^50^, ^51^ HCPs should discuss with their patients the risks of cannabis co‐use with tobacco and alcohol, and provide individualized guidance on the best route of administration that minimizes behavioral risks. Furthermore, our finding of a lower likelihood of awareness among male survivors aligns with a previous study that found increased use of cannabis among female patients with cancer.^52^ The greater awareness of cannabis use in cancer management among female survivors in our study may be related to the sex‐based differences in symptoms and functioning among survivors.^31^ Compared to their male counterparts, female survivors are more prone to experiencing severe side effects from cancer treatment and exhibit greater sensitivity to pain—a prevalent symptom associated with cancer and its treatment.^53^, ^54^, ^55^ Hence, female survivors are likely to be aware of alternate approaches, such as medicinal cannabis, as they search for different means to alleviate their cancer and treatment‐related symptoms. Overall, this finding highlights the need for gender‐tailored educational interventions to raise awareness of the potential benefits and harms of cannabis for cancer management among survivors. Overall, the prevalence of awareness of cannabis use in cancer management among survivors was higher than the prevalence of interest in learning about cannabis use for cancer management across the different patient characteristics assessed. This difference in level of awareness relative to interest in cannabis use for cancer management could be due to several factors. Whereas awareness could influence action, awareness may not always result in interest or action in health behavior. Moreover, personal and social factors such as perceived stigma, religious beliefs, state laws criminalizing medicinal cannabis, and barriers to cannabis access and procurement may discourage survivors who are aware of cannabis use from indicating interest in cannabis use for cancer management.^56^, ^57^, ^58^, ^59^

This study has few limitations. Most patients attending MDACC are generally of higher economic status and are predominantly urban dwellers, limiting the generalizability of our results. As such, findings from our study should be interpreted in the context of the sociodemographics of survivors included in our study. Also, given that our survey was anonymous, we were unable to connect respondents to additional clinical parameters. However, the anonymous nature of our survey increases the validity of participants self‐report on cannabis use for cancer management. Although our study had a low response rate, we recalibrated the survey design weight to account for nonresponse bias, thus increasing the generalizability of our findings. Furthermore, this is the largest study of cancer survivors to have examined the source of information for cannabis use in cancer management and the factors that predict awareness of and interest in cannabis use for cancer management.

In conclusion, most cancer survivors are aware of and interested in cannabis use for medical management. However, many survivors still seek information on cannabis use for cancer management from nonscientific and untrusted sources. Whereas non‐Hispanic Blacks were less aware of and interested in cannabis use for cancer management, those supporting cannabis legislation were more likely to be aware of and interested in cannabis use for cancer management. Also, survivors in active treatment were more likely to be interested in cannabis use for cancer management. This study highlights the need to regularly assess patients' awareness and interest in the use of cannabis products for cancer management and to tailor educational interventions and HCP communications on possible uses and risks of cannabis in order to prevent misinformation and potential toxicities. Future studies should evaluate the efficacy of health care providers in discussing cannabis use in cancer management with survivors. Also, longitudinal studies to comprehensively assess the benefits and risks of cannabis use during and after cancer treatment among adult survivors are needed.

## AUTHOR CONTRIBUTIONS

**Ikponmwosa Osaghae:** Conceptualization (equal); methodology (equal); writing – original draft. **Rajesh Talluri:** Data curation (equal); formal analysis; methodology (equal); writing – review and editing (equal). **Onyema Greg Chido‐Amajuoyi:** Conceptualization (equal); methodology (equal); writing – review and editing (equal). **Kimberson Tanco:** Writing – review and editing (equal). **Dimpy P. Shah:** Writing – review and editing (equal). **Mala Pande:** Conceptualization (equal); methodology (equal); writing – review and editing (equal). **Sanjay Shete:** Conceptualization (equal); data curation (equal); funding acquisition; methodology (equal); project administration; supervision; writing – review and editing (equal).

## FUNDING INFORMATION

The study was funded by the National Cancer Institute (P30CA016672 to S. Shete), the Betty B. Marcus Chair in Cancer Prevention (to S. Shete), the Duncan Family Institute for Cancer Prevention and Risk Assessment (S. Shete), and the Cancer Prevention Research Institute of Texas (Grant RP170259 to S. Shete). The funders were not involved in the study design, analysis, interpretation of data, or manuscript writing.

## CONFLICT OF INTEREST STATEMENT

All authors have no conflict of interests relevant to this article to disclose.

## Data Availability

Data are available from the corresponding author on reasonable request.
